# The persistence of naturally acquired antibodies and memory B cells specific to rhoptry proteins of *Plasmodium vivax* in patients from areas of low malaria transmission

**DOI:** 10.1186/s12936-019-3009-2

**Published:** 2019-11-29

**Authors:** Piyawan Kochayoo, Siriruk Changrob, Kittikorn Wangriatisak, Seong Kyun Lee, Patchanee Chootong, Eun-Taek Han

**Affiliations:** 1 Department of Clinical Microbiology and Applied Technology, Faculty of Medical Technology, Mahidol University, Bangkok, 10700 Thailand; 2 Department of Medical Environmental Biology and Tropical Medicine, School of Medicine, Kangwon National University, Chuncheon, Gangwon-do 200-701 Republic of Korea

**Keywords:** *Plasmodium vivax*, PvRALP1-Ecto, PvRhopH2, Antibody responses, Memory B cells

## Abstract

**Background:**

Rhoptries are the large, paired, secretory organelles located at the apical tip of the malaria merozoite that are considered important for parasite invasion processes. *Plasmodium vivax* rhoptry proteins have been shown to induce humoral immunity during natural infections. Therefore, these proteins may be potential novel vaccine candidates. However, there is a lack of data on the duration of antibody and memory B cell (MBC) responses. Here, the longitudinal analysis of antibody and MBC responses to the *P. vivax* rhoptry proteins PvRALP1-Ecto and PvRhopH2 were monitored and analysed in individuals to determine their persistence.

**Methods:**

Thirty-nine samples from *P. vivax*-infected subjects (age 18–60 years) were recruited to explore the frequency and persistence of antibody and MBC responses against rhoptry proteins (PvRALP1-Ecto and PvRhopH2) using both cross-sectional and longitudinal cohort study designs. Antibody levels were determined by ELISA during clinical malaria, and at 3, 9 and 12 months post-infection. The frequency of MBC sub-sets and presence of rhoptry-specific MBCs in subjects 18 months after treatment were detected by flow cytometry and ELISPOT assay.

**Results:**

The seroprevalence of antibodies against PvRALP1-Ecto and PvRhopH2 proteins was found to be high during acute infection, with IgG1, IgG2 and IgG3 sub-classes predominant. However, these anti-rhoptry responses were short-lived and significantly decreased at 9 months post-infection. To relate the durability of these antibody responses to MBC persistence at post-infection, 18-month post-infection peripheral blood mononuclear cells (PBMCs) samples were taken to detect rhoptry-specific MBCs and frequency of MBC sub-sets, and correlate with antibody responses. These late post-infection samples revealed that rhoptry-specific MBCs were present in about 70% of total subjects. However, the persistence of specific MBCs was not correlated with antibody responses as the majority of malaria subjects who were positive for PvRALP1-Ecto- or PvRhopH2-specific MBCs were seronegative for the rhoptry antigens. The frequencies of classical MBCs were increased after infection, whereas those of activated and atypical MBCs were decreased, indicating that MBC responses could switch from activated or atypical MBCs to classical MBCs after parasite clearance, and were maintained in blood circulating at post-infection.

**Conclusion:**

The study showed that rhoptry antigens induced the development and persistence of MBC responses in *P. vivax*-infected subjects who lived in a region of low malaria transmission, which were not related to the longevity of antibody responses.

## Background

Malaria remains an important global health issue causing illness and death worldwide, especially in tropical and sub-tropical areas. There were an estimated 219 million malaria cases and more than 400,000 deaths in 2017 [1]. *Plasmodium vivax* is the most prevalent cause of recurring malaria and has a wide geographic distribution, mainly in Asia–Pacific and Latin American regions [2]. Relapse is a unique feature of *P. vivax* biology, making it difficult to control because the parasite can be hidden for a long time in the patient’s liver and then emerge to cause relapsing *P. vivax* [3, 4]. Given the difficulties with treatment, diagnosis and control, an effective vaccine would be valuable in eliminating and preventing the disease [4].

There is currently no licensed vaccine available for malaria. Several antigen candidates against *Plasmodium falciparum* have moved into clinical trials. In contrast, *P. vivax* vaccine development has lagged far behind, as only three *P. vivax* vaccine candidates (PvDBP, PvCSP, Pvs25) have even reached phase I clinical trials [5–7]. This may reflect previous neglect of *P. vivax*, technical challenges such as maintaining *P. vivax* in culture, and limited animal models of infection. For blood-stage malaria vaccines, the aim is to block parasite invasion and subsequently reduce the clinical burden. At present, *P. vivax* Duffy-binding protein region II (PvDBPII) is a leading vaccine candidate because *P. vivax* invasion of erythrocytes is largely dependent upon its interaction with the Duffy blood-group antigen [8]. It induces antibody responses in populations naturally exposed to *P. vivax* and protects against high-density *P. vivax* infection by inhibiting parasite invasion into red blood cells (RBCs) [9]. However, high polymorphism of this micronemal protein is a major challenge in designing a vaccine that will produce protective immunity responses against the conserved epitopes against a panel of variant *P. vivax* isolates [10]. Moreover, vivax malaria in Duffy-negative individuals in Africa was recently reported, indicating that there are Duffy antigen/chemokine receptor (DARC)-independent pathways for *P. vivax* invasion [11]. Thus, a novel parasite ligand is required for parasite invasion. Such a protein, which has immunogenicity to elicit immune responses that will block merozoite invasion of RBCs and stop rapid replication of merozoites, has been identified.

Antibody responses to blood-stage malaria are required for inhibition of parasite invasion [12–15]. Longitudinal studies of humans living in areas of high malaria transmission showed that repeated *P. falciparum* infections can induce antibody responses to blood-stage antigens but that these responses were relatively short-lived [16]. Likewise, the antibody profiles in malaria-naïve and semi-immune Colombian volunteers experimentally infected with *P. vivax* were short-lived and had returned to near baseline by day 145 [17]. The presence of these antibody responses was independent of the presence of malaria-specific memory B cells (MBCs), since individuals residing in areas of low transmission have been shown to generate stable *Plasmodium*-specific MBCs without frequent boosting [18, 19]. However, children and young adults in an area of high seasonal transmission in Mali demonstrated delayed development of *P. falciparum*-specific MBCs despite repeated infections annually [20, 21]. This evidence indicates that infection rates and the diverse genetics of both parasite and host could be involved in the development and maintenance of malaria-specific MBCs [22–24]. Therefore, better understanding of the durability of both antibody and MBC responses in patients with naturally acquired malaria, as well as identifying the antigens that induce these long-term responses, will be useful in the development of protective blood-stage malaria vaccines.

The rhoptry is a large secretory organelle of the merozoite that contains a wide range of proteins which are involved in invasion of RBCs [25, 26]. It is composed of at least two sub-domains, referred to as the rhoptry neck and the rhoptry bulb. Several rhoptry proteins, such as *P. falciparum* rhoptry neck protein (RON) 2–4, are involved in tight junction formation between the parasite and its target cells by interacting with the micronemal protein apical membrane antigen (AMA) 1 [27, 28]. However, some rhoptry proteins are released during invasion and migrate to the lumen or membrane of the parasitophorous vacuole [29]. The rhoptry-associated leucine zipper-like protein 1 (RALP1) and high-molecular-weight complex rhoptry proteins (RhopH) have been characterized as being crucial during *P. falciparum* infection [30, 31]. These two proteins are localized in the rhoptry of *P. falciparum* merozoites [30, 32]. RALP1 possesses a leucine zipper-like domain that facilitates protein–protein interaction [30] and RhopH2 contains a signal peptide at its N-terminal and 12 cysteines in its C-terminal half [33]. Furthermore, these rhoptry proteins are conserved in *Plasmodium* spp. [34], suggesting that they may be involved in parasite invasion.

High antigenicity of rhoptry proteins has often been reported in malaria patients [33, 35–37]. The rhoptry neck protein of merozoites, PvRALP1, triggers IgG3, IgG2 and IgG1 isotype responses in *P. vivax*-exposed Korean individuals, and purified serum anti-PfRALP1 from Malian *P. falciparum*-infected patients inhibits parasite invasion [30, 35]. Two *P. vivax* proteins at the rhoptry bulb, rhoptry-associated membrane protein (RAMA) and RhopH2 antigens, show an ability to induce the acquisition of humoral immunity in both mouse models and patients [33, 38]. Interestingly, the anti-PvRAMA response is maintained up to 9 months after anti-malarial treatment, and some patients maintain antibody responses up to 12 months post-infection [38]. All of these data indicate that the combination of these rhoptry proteins with other blood-stage proteins in a vaccine design may induce protective responses of humoral immunity. However, due to poor understanding of the immune responses to rhoptry proteins and the duration of these responses, more data are required from immuno-epidemiological studies on naturally acquired MBCs and antibody responses. Thus, in the present study two rhoptry protein antigens, PvRALP1-Ecto and PvRhopH2, were used to characterize the long-term responses of antibodies and MBCs in *P. vivax* patients living in a low-malaria-endemicity region of southern Thailand. Longitudinal sampling of participants in a cohort study was conducted to determine whether individuals exposed to *P. vivax* developed and were able to maintain antibody and MBC responses to these rhoptry proteins.

## Methods

### Study site and sample collection

This study was conducted in low-malaria-transmission areas of southern Thailand (Chumphon Province) where both *P. falciparum* and *P. vivax* were common. Both a cross-sectional survey and a cohort study were set up between 2014 and 2017 to explore the antigenicity of rhoptry proteins and monitor the longevity of antibody and MBC responses. First, serological responses to rhoptry proteins were determined from 39 patients with acute *P. vivax* infection (Table 1). To observe the development and maintenance of antibody and MBC responses, only individuals with samples available from all follow-up time points were used (18 subjects; Table 2). Subsequently, the samples were analysed for total IgG and subtype IgG as detected by ELISA (sub-cohort 1 study).

**Table 1:** Characteristics of study participants in survey of PvRhoptry antigenicity during acute *Plasmodium vivax* infection

Characteristic	Acute vivax patients	Healthy subjects
Total (n)	39	27
Gender		
Male	27	8
Female	12	19
Ages (years) Mean ± SD (range)	40.1 ± 11.4 (18–60)	20.2 ± 0.8 (19–21)
Seropositive responders		
PvRALP1-Ecto	26	0
PvRhopH2	29	0
Both PvRALP1 and PvRhopH2	22	0
Parasitaemia (parasite/µL) Mean ± SD (range)	5170.8 ± 5238.3 (200–15,000)	0

**Table 2:** Characteristics of participants (n = 18) in the study of total IgG and IgG isotype longevity

By antigen	No. positive responders at acute phase	Age	Gender		Nationality		No. recorded re-infections
		Mean ± SD	Male	Female	Thai	Myanmar	
Pv-RALP1-Ecto	14	44.2 ± 9.7	13	5	17	1	1
PvRhopH2	14	44.2 ± 9.7	13	5	17	1	1

To explore the basis of persistence of humoral immunity against rhoptry antigens in *P. vivax* infection, the kinetics of B cell sub-sets in the acute and recovery phases, and the persistence of rhoptry-specific MBCs were investigated in the sub-cohort 2 study (May 2016 to May 2018). Peripheral blood mononuclear cells (PBMCs) from 10 individuals were isolated to perform for flow-cytometric analysis and ELISPOT assays. Plasma was collected for detection of total IgG and IgG subclasses. Peripheral blood samples (10 mL) from participants were obtained by venipuncture into heparinized tubes before administration of treatment, and transported to the laboratory for processing within 6 h of collection. The PBMCs were separated by Ficoll-Hypaque density-gradient centrifugation and used for MBC experiments. Plasma was stored at − 20 °C for antibody analyses.

The inclusion criteria for subject recruitment were the following: (1) systolic blood pressure not less than 90 mmHg; (2) body temperature not higher than 40 °C; (3) haematocrit not less than 25%; (4) no prior treatment with corticoids or non-steroidal anti-inflammatory drugs (NSAIDs); and, (5) age of 18 years or above. Those who did not meet the criteria were excluded. Written informed consent was obtained from all participants before enrollment. The *P. vivax* infection was diagnosed by microscopic examination of both thin and thick blood films, and confirmed by nested PCR. Data on occurrence and number of prior *P. vivax* infections in each individual were obtained from the records of the local Vector-borne Disease Unit 11.4.2. The malaria staff collected post-treatment blood samples from patients every 3 months to check for sub-clinical malaria by nested PCR. To estimate the incidence of clinical malaria over the study period, malaria staff had carried out weekly house-to-house visits from May 2014 to May 2017, and in May 2018, with confirmation of infection by nested PCR. Malaria-unexposed persons living in Bangkok, Thailand, were recruited as healthy controls (HC, n = 27). Ethical approvals were obtained from the Committee on Human Rights Related to Human Experimentation, Mahidol University, Thailand (MUIR 2012/079.2408).

### Expression and purification of recombinant PvRALP1-Ecto and PvRhopH2 proteins

To express the recombinant PvRALP1-Ecto and PvRhopH2 proteins, the protocol followed that of previous studies [33, 35]. In brief, the open reading frame of *pvralp1*-*ecto*, comprising amino acids 31 to 675 without the signal peptide sequence, and the first exon of the *pvrhoph2* sequence, amino acid position 67 to 1161, were amplified with the following primers: PvRALP1-Ecto F: 5′-atcactagttctcgagATGGCGTACCGCCTAAAGAGG-3′ and the reverse primer PvRALP1-Ecto R: 5′-ccctatatatggatccTCACTAGAACATGTCGTAGAGCAGGC-3′, RhopH2 F:PvRhopH2 F: 5′-GGGCGGATATCTCGAGGAGCTGAGCCACAGCTTGT-3′ and PvRhopH2 R: 5′-GCGGTACCCGGGATCCTCACTTCTCCACATCCTCGTGGT-3′. The purified PCR products were then cloned into the *Xho*I and *BamH*I sites of the pEU-E01-His-Tev-N2 plasmid vector, which is an expression plasmid with an N-terminal hexa-histidine (His)-tag followed by a tobacco etch virus protease cleavage site. Plasmid DNA was prepared, purified and eluted in 0.1× TE buffer (10 mM Tris–HCl, pH 8.0, 1 mM EDTA), and used for in vitro transcription. The recombinant proteins were synthesized with a wheat germ cell-free protein expression system (CellFree Sciences, Matsuyama, Japan), using the bilayer translation reaction method as described previously [39]. The His-tagged recombinant proteins were purified using a Ni-Sepharose column (GE Healthcare, Camarillo, CA, USA) and eluted with phosphate-buffered saline (PBS) containing 500 nM imidazole. Finally, the purified recombinant proteins PvRALP1-Ecto and PvRhopH2 were detected by SDS-PAGE and Western blot analyses.

### Measurement of total IgG and IgG subtype responses

Naturally acquired antibodies against recombinant PvRALP1-Ecto and PvRhopH2 were detected by ELISA. In brief, 96-well, flat-bottom microplates (Corning, Corning, NY, USA) were pre-coated with 0.5 μg recombinant PvRALP1-Ecto or PvRhopH2 per well and incubated overnight at 4 °C. After blocking with PBS containing 4% BSA for 2 h, 100 μL of each plasma sample at a dilution of 1:200 was added to duplicate wells and incubated at room temperature for 1 h. The plates were washed with PBS containing 0.05% Tween 20 (PBS-T) five times and incubated with peroxidase-conjugated, goat anti-human IgG (Sigma, St. Louis, MO, USA) for 1 h. Subsequently, washing was repeated, and the plates were developed with 2,2′-azino-bis (3-ethylbenzthiazoline-6-sulphonic acid) substrate (R&D Systems, Minneapolis, MN, USA) for 45 min. The optical density (OD) was determined at 405 nm using a plate reader. The positive samples (n = 2) were *P. vivax* plasma that showed seropositive to rDBPII antigen from previous study [40]. The OD values for duplicate wells per individual were averaged. A baseline OD was established using plasma samples from 27 healthy individuals living in non-malaria endemic areas (HC).

IgG isotype responses to rhoptry proteins were detected following a protocol similar to that for total IgG detection. In the step when each plasma sample was added at a dilution of 1:100, followed by addition of conjugated antibodies, horseradish peroxidase (HRP)-conjugated anti-human IgG1, IgG2, IgG3, and IgG4 antibodies at a dilution of 1:1000 were used for detection. The OD of the reaction was measured at 450 nm after the addition of the tetramethylbenzidine (TMB) enzyme–substrate. The positive controls were plasma (n = 2) of the vivax malaria patient who had the highest OD value for total IgG. A baseline OD was established using plasma samples from 20 healthy individuals living in non-malaria endemic areas (HC).

The levels of total IgG or IgG subclasses specific to PvRALP1-Ecto or PvRhopH2 were expressed as reactivity indexes (RI), which were obtained by dividing the mean sample OD by the cut-off value. The cut-off value (mean OD + 2 standard deviations (SDs)) was calculated from a set of negative controls obtained from plasma samples from HC. A sample was considered to be positive for a specific antibody if the RI was ≥ 1.

### Memory B cell phenotyping

PBMCs collected during acute malaria and 18 months post-infection were used for B cell sub-set phenotyping. Fluorochrome-conjugated, mouse anti-human monoclonal antibodies were used to stain 1 million PBMCs/100 μL FACS buffer. A cocktail consisting of the following mouse monoclonal antibodies was used: FITC-CD19, PerCP-CD20, PE/Cy7-CD10, PE-CD27, and APC-CD21 (Biolegend, San Diego, CA, USA). After staining for 15 min, cells were washed with FACS buffer. Finally, cells were suspended in 250 μL FACS buffer. The analyses were done by a flow cytometer (BD FACSCanto II, Becton–Dickinson Immunocytometry Systems, San Jose, CA, USA). Data were processed using FlowJo software (Tree Star, San Carlos, CA, USA).

### B cell ELISPOT assay

The presence of rhoptry-specific MBCs was determined by ELISPOT assay as performed in a previous study [41]. In brief, 9 × 10^6^ PBMCs from *P. vivax* subjects were prepared for cell stimulation along with negative controls. First, 1 × 10^6^ cells per mL were stimulated with polyclonal activator R848 and recombinant human IL-2 (Mabtech AB, Stockholm, Sweden) at 37 °C in a humidified 5% CO_2_ incubator for 72 h. Then, the ELISPOT assay was performed by coating with 15 μg/mL of monoclonal antibodies of anti-human IgG (clones MT91/145; Mabtech), 5 μg/mL of recombinant PvRALP1-Ecto or PvRhopH2 rhoptry antigens, or 1 μg/mL of tetanus toxoid (TT) antigen (Merck Millipore, Darmstadt, Germany) onto MultiScreen Filter PVDF Immobilon plates (Merck Millipore). Cells were harvested and seeded in duplicate to yield 5 × 10^4^ cells per anti-human IgG-coated well, and 1 × 10^6^ cells per specific antigen-coated well. After 24 h of incubation at 37 °C under 5% CO_2_, 1 μg/mL of detection monoclonal antibodies MT78/145 (Mabtech) diluted in PBS-0.5% FBS was added and incubated for 2 h at room temperature. Following a thorough washing with PBS, the diluted (1:1000) streptavidin-HRP-conjugated, polyclonal goat anti-human IgG (Mabtech) and TMB substrate for ELISPOT (Mabtech) were added. The filtered plates were rinsed with deionized water after distinct spots emerged. Antigen-specific MBCs were expressed as spot-forming cells (SFCs) in the wells. The plates were analysed with a Bioreader 5000 Pro-F gamma ELISPOT Reader (BioSys GmbH, Karben, Germany). PBMCs from *P. vivax*-infected subjects that were cultured without stimulation and incubated overnight with rhoptry antigens were used as negative controls. A positive ELISPOT response was defined as detectable spots in duplicate wells with the total spots in the specific antigen-coated wells being at least twice the number of spots detected with the negative control samples.

### Statistical analyses

The Mann–Whitney U test was used to compare data of 2 groups of samples (unpaired) from different subjects, while the Wilcoxon signed rank test was used to compare data of 2 groups of paired data from the same subjects at different time points. Kruskal–Wallis one-way ANOVA was used to compare data from more than 2 groups. The association between the responses of rhoptry-specific MBCs by ELISPOT and antibodies by ELISA was done using Fisher’s exact tests. In all analyses, 2-tailed *P*-values < 0.05 were considered significant. The statistical analysis was performed and graphs prepared using GraphPad Prism software (Systat Software, San Jose, CA, USA).

## Results

### Sample organization

**Fig. 1:**
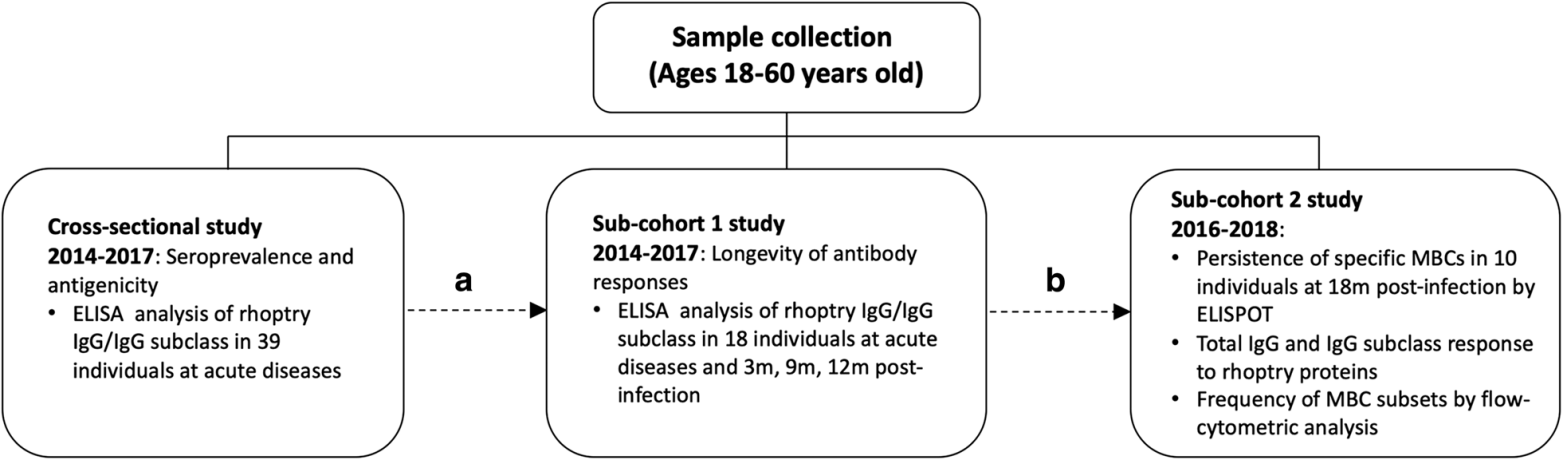
Flow chart of the sample organization in this study. Each dashed line refers to the sample which was taken for further experimentation. **a** Subjects who had complete follow up at all time points of cohort study. **b** Subjects who had PBMCs available for flow cytometric analysis and ELISPOT assay, and plasma available for ELISA technique at 18 months post-infection

There are three distinct groups of data in this study (Fig. 1). First, the cross-sectional survey was conducted in 39 *P. vivax*-infected patients, using plasma samples collected from May 2014 to May 2017 (Table 1). Some of these individuals dropped out of some sample collections. From the total 39 infected subjects, blood samples were available at all time points from 18 subjects. These 18 subjects were studied as ‘sub-cohort 1’ to explore the longevity of antibody responses (both total IgG and IgG isotype levels) against PvRALP1-Ecto and PvRhopH2 antigens (Table 2). Furthermore, to assess the relationship between persistence of detectable antibody and MBC responses post-infection, ‘sub-cohort 2’ was created by recruiting 10 *P. vivax*-infected patients between May 2016 and May 2018 to determine MBC sub-set responses and the persistence of PvRALP1-Ecto- and PvRhopH2-specific MBCs at 18 months post-infection.

### Antigenicity of rhoptry antigens in acute and recovery phases of *Plasmodium vivax* infection

**Fig. 2:**
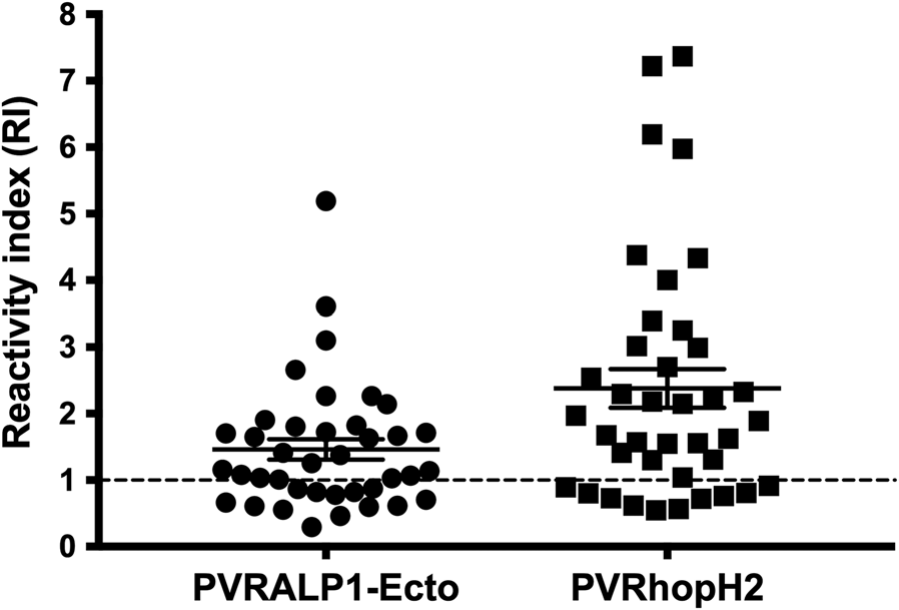
The seroprevalence of anti-IgG rhoptry responses. The reactive index (RI) of specific IgG responses to PvRALP1-Ecto and PvRhopH2 antigens in the plasma of acute *P. vivax*-infected patients (n = 39) was detected ELISA. The horizontal line represents mean values ± SEM. Dashed line indicates the cut-off value (RI ≥ 1)

To survey the antigenicity of rhoptry proteins in natural exposure to *P. vivax* parasites, the antibody titres against recombinant PvRALP1-Ecto and PvRhopH2 antigens were determined during infection using a cross-sectional survey. In the acute phase, anti-rhoptry responses were significantly higher than those in healthy controls (PvRALP1-Ecto; P < 0.0001, PvRhopH2; P < 0.0001). Subject positivity to PvRALP1-Ecto and PvRhopH2 was 66.67% (26 of 39) and 29 74.36% (29 of 39), respectively (Fig. 2). Among PvRALP1-Ecto and PvRHopH2 seropositive responders, there were 22 patients who were antibody positive against both rhoptry proteins (Table 1). Thus, the longevity of antibody responses against rhoptry proteins was further explored in the sub-cohort 1 study.

### Persistence of anti-rhoptry IgG specific responses after acute infection with *Plasmodium vivax*

**Fig. 3:**
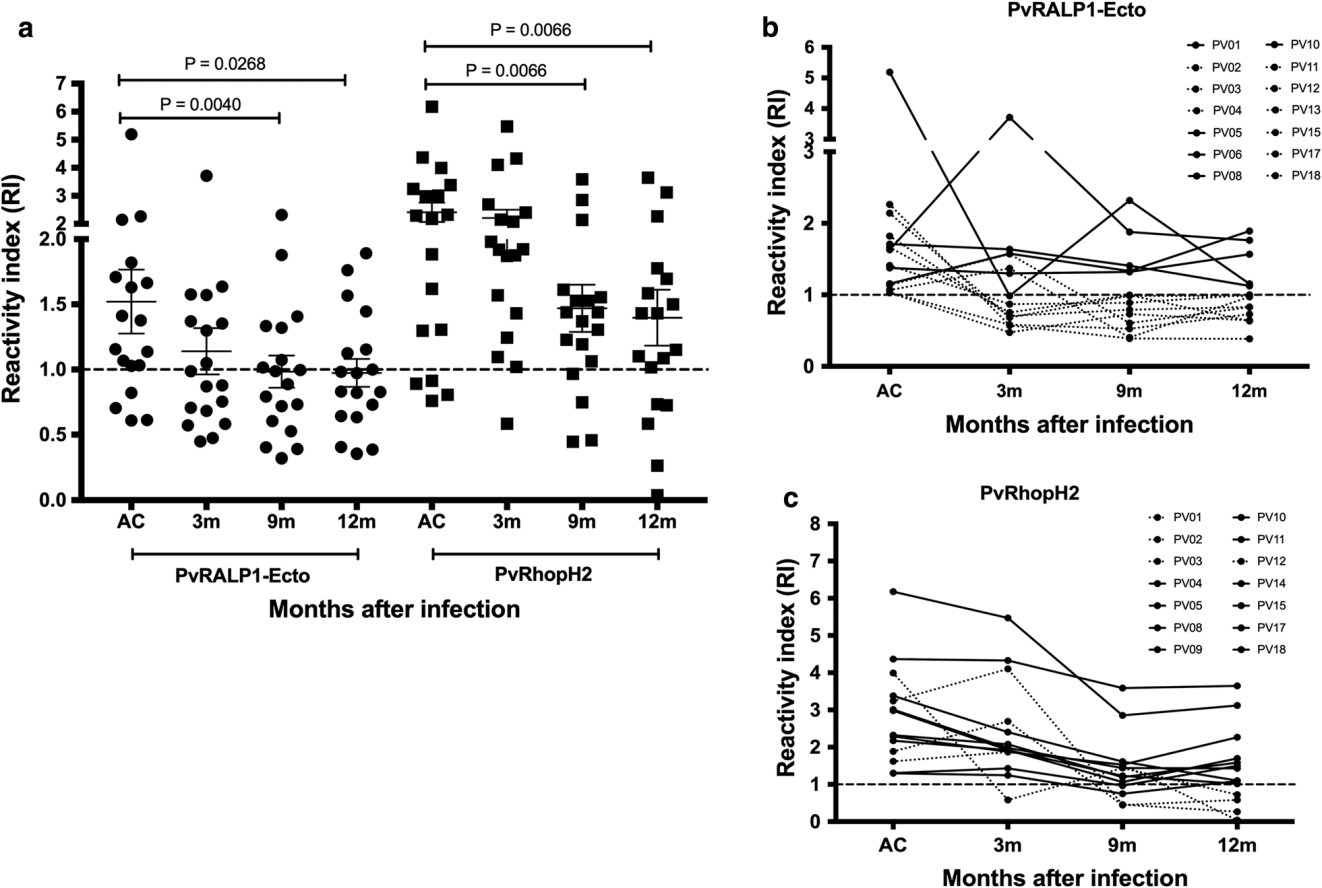
Longitudinal analysis of anti-rhoptry responses. The reactive index (RI) of specific IgG responses to PvRALP1-Ecto and PvRhopH2 antigens in the plasma of *P. vivax* subjects during follow-up was detected by ELISA. **a** Anti-PvRALP1-Ecto and anti-PvRhopH2 responses at all time points in the cohort study: acute infection and 3, 9, and 12 months after infection (n = 18). The horizontal line represents mean values ± SEM. **b**, **c** The kinetics of antibody responses against PvRALP1-Ecto and PvRhopH2 in 14 seropositive *P. vivax*-infected patients at different time points: acute infection and 3, 9, and 12 months after infection. Solid lines indicate individual subjects who have antibody responses to PvRALP1-Ecto and PvRhopH2 up to 12 months. Dotted lines indicate individual subjects who have antibody responses to PvRALP1-Ecto and PvRhopH2 less than 12 months. Dashed line indicates the cut-off value (RI ≥ 1)

To determine the durability of anti-rhoptry responses after infection, antibody titres were measured in the 18 individuals who were monitored at all time points of the cohort study (acute, 3, 9 and 12 months). Both anti-PvRALP1-Ecto and anti-PvRhopH2 titres were significantly reduced at 9 months post-infection (Fig. 3a). Only 38.89 and 77.78% of patients showed a positive response to PvRALP1-Ecto and PvRhopH2 antigens, respectively, at 9 months post-infection (Fig. 3a). Interestingly, the monitoring kinetic of PvRALP1-Ecto and PvRhopH2 responses up to 12 months’ post-infection showed that 5 and 10 patients, respectively, stably maintained their antibody responses (Fig. 3b, c). Thus, the IgG isotypes which played major roles in the humoral immunity against rhoptry protein was further explored in acute vivax malaria and after infection.

### IgG2 isotype had a stable response post-infection

**Fig. 4:**
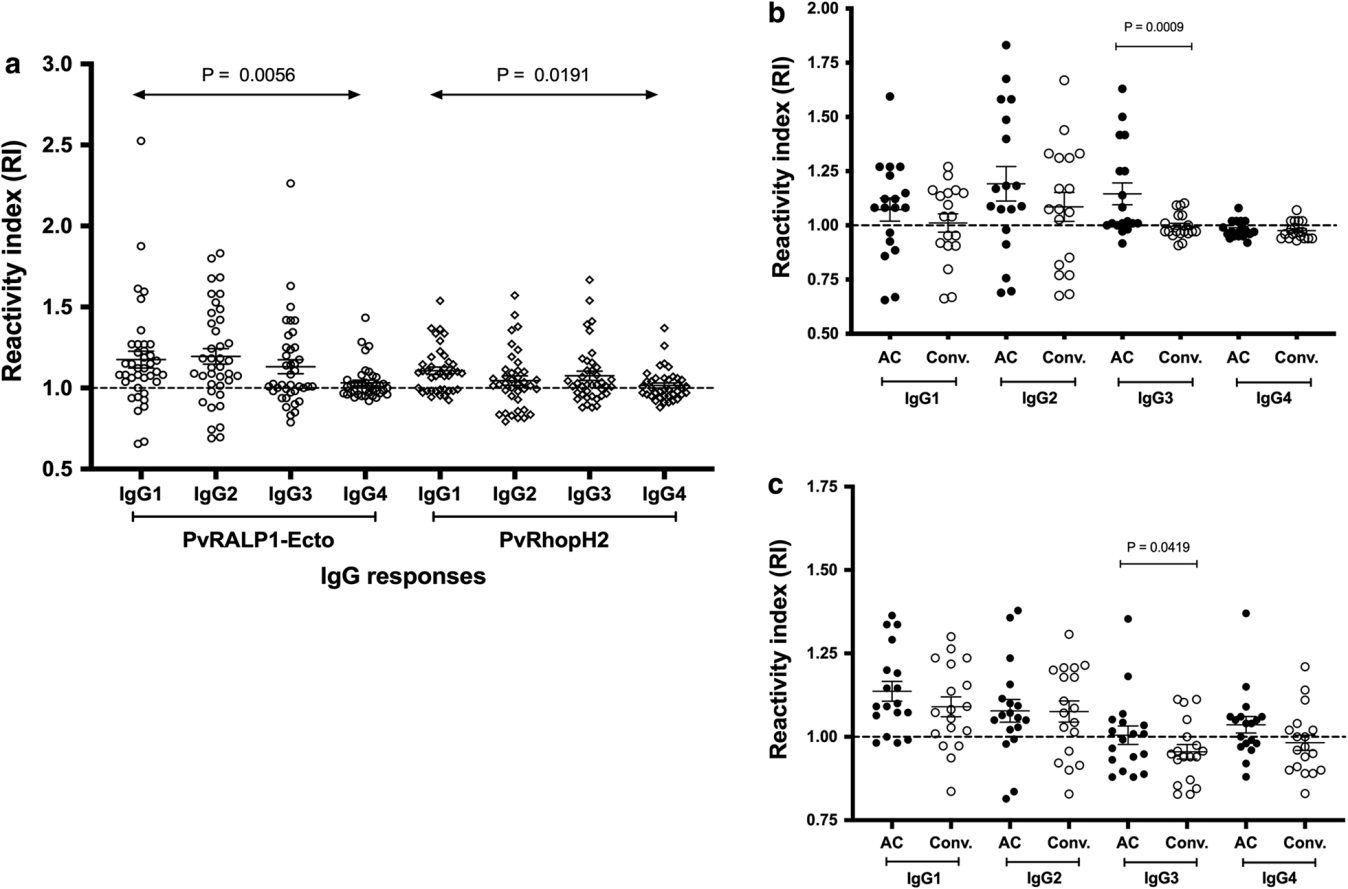
The prevalence of IgG sub-class responses to rhoptry proteins. The reactive index (RI) of specific IgG sub-class responses to PvRALP1-Ecto and PvRhopH2 antigens in the plasma of *P. vivax* subjects at acute and during follow-up was detected by ELISA. **a** The comparative analysis of anti-IgG1, -IgG2, -IgG3, and -IgG4 specific to PvRALP1-Ecto and PvRhopH2 proteins in individuals with acute *P. vivax* malaria (AC, n = 39). The Kruskal–Wallis one-way ANOVA test was performed to compare the mean antibody levels of the difference IgG sub-classes. The horizontal line represents mean values ± SEM. **b**, **c** The anti-IgG1, -IgG2, IgG3 and -IgG4 responses to these two rhoptry proteins in 18 *P. vivax* subjects in the acute phase (AC) and convalescence phase (Conv.; 9 months post-infection). The comparisons of IgG sub-class response in the plasma of subjects during follow up (AC and Conv., n = 18) were done by Wilcoxon signed rank tests. Dashed line indicates the cut-off value (RI ≥ 1)

As different IgG sub-classes have the ability to provide protective immunity [42, 43], the predominant anti-IgG rhoptry antibody isotype was determined in the acute and recovery phases of these *P. vivax* infections. In the acute phase, the prevalent rates of anti-PvRALP1-Ecto IgG sub-classes were 79.49% for IgG1, 76.92% for IgG2, 69.23% for IgG3, and 53.85% for IgG4. In case of anti-PvRhopH2 IgG subclass responses, the IgG1, IgG2, IgG3, and IgG4 frequency distribution of individuals that recognized the antigen was 64.10%, 58.97%, 66.67%, and 43.59%, respectively (Fig. 4a). Therefore, the predominant sub-class response to PvRALP1-Ecto was IgG1 and IgG2 whereas a higher seroprevalence of IgG1 and IgG3 responders was shown in PvRhopH2. A further analysis of IgG isotype responses during acute malaria and 9 months post-infection in sub-cohort 2 subjects showed that IgG1 and IgG2 response to PvRALP1-Ecto at 9 months post-infection was not significantly different compared to those during the acute phase (Fig. 4b). However, a greater decrease of IgG3 responses to these two rhoptry protein was shown at 9 months post-infection (Fig. 4b, c).

### Memory B cells specific to rhoptry proteins were detected at least 18 months post-infection

**Fig. 5:**
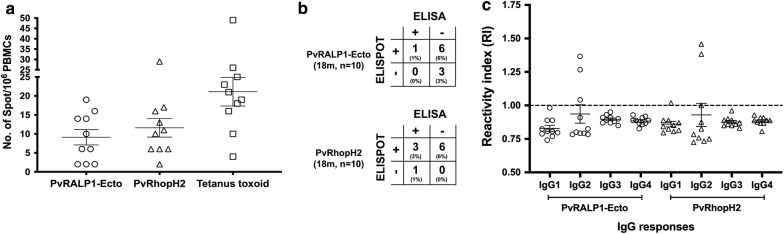
Rhoptry-specific MBCs detected 18 months post-infection. **a** The number of specific MBC responses to the PvRALP1-Ecto and PvRhopH2 antigens, and tetanus toxoid (TT) in PBMCs from subjects 18 months after *P. vivax* infection were determined by ELISPOT (n = 10). The frequency of MBCs was expressed per million cultured PBMCs. Each symbol represents the MBC number for one individual. The line reflects the median value. **b** The correlation analysis between the responses of rhoptry-specific MBCs and antibody in 10 subjects at 18 months post-infection. **c** The comparative analysis of anti-IgG1, -IgG2, -IgG3 and -IgG4 specific to PvRALP1-Ecto and PvRhopH2 proteins in 10 subjects at 18 months post-infection. The Kruskal–Wallis one-way ANOVA test was performed to compare the mean antibody levels of the difference IgG subclasses. The horizontal line represents mean values ± SEM. Dashed line indicates the cut-off value (RI ≥ 1)

As some patients showed the persistence of anti-rhoptry antibodies for at least 12 months, this could be due to the ability of rhoptry antigens to induce MBC development during acute malaria and to maintain it after recovery from infection. The sub-cohort 2 study was designed to explore the presence of rhoptry-specific MBC responses during the recovery phase (see Additional file 1). Of the 10 subjects, 7 and 9 had MBCs to PvRALP1-Ecto and PvRhopH2 antigens, respectively (Fig. 5a). The PvRALP1-Ecto- and PvRHopH2-specific MBCs showed spot forming cells (SFCs) in the ranges of 2–19 and 2–29, respectively. As expected, all patients produced TT-specific MBCs (range, 4–49 SFCs). A statistical analysis of association between the responses of rhoptry-specific MBCs and antibodies in individuals at 18 months post-infection showed that of 7 individuals positive for MBCs to PvRALP1-Ecto, 6 were seronegative (P value = 1.00); of 9 individuals positive for MBCs to PvRhopH2, 6 were seronegative (P value = 0.40) (Fig. 5b). In addition, the seronegative levels of anti-PvRALP1-Ecto and -PvRhopH2 IgG were related with the short-lived of IgG sub-class response at 18 months post-infection patients. Of total 10 samples, only 3, 1 and 2 patients maintained seropositive for anti-PvRALP1-Ecto-IgG2, anti-PvRhopH2-IgG1 and anti-PvRhopH2-IgG2 responses, respectively (Fig. 5c).

### B cell surface marker analysis showed functional of MBC responses

**Fig. 6:**
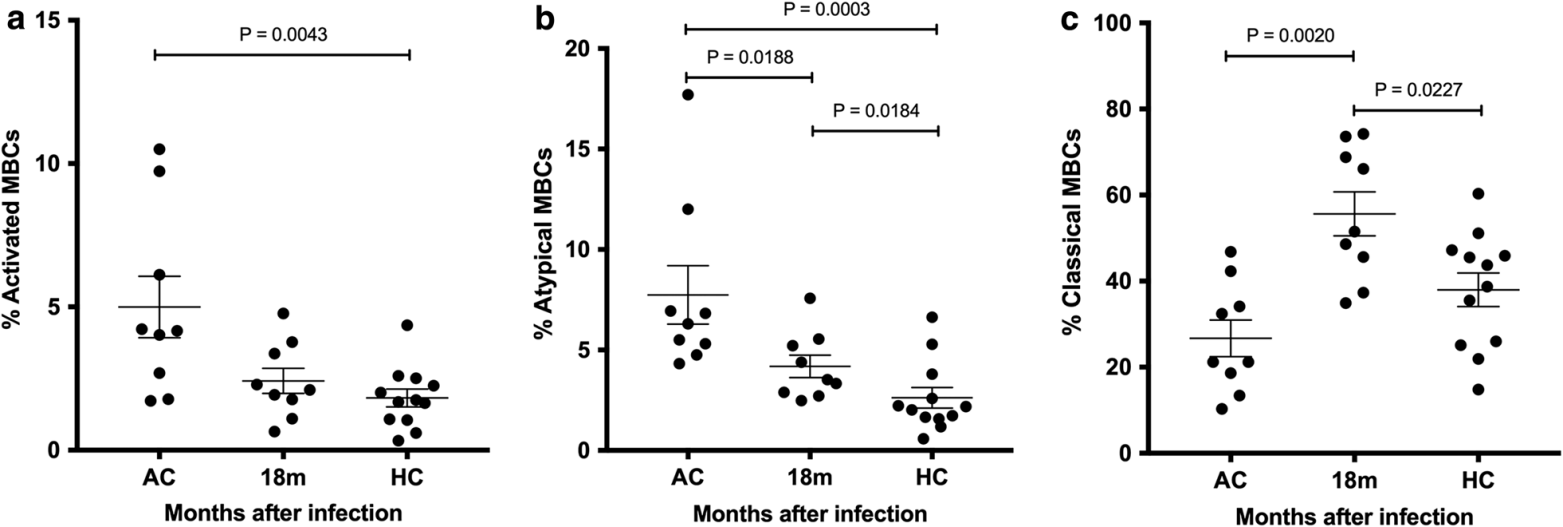
The frequency of MBC subsets during acute and recovery (18 months post-infection) phases. The frequencies of (**a**) activated, (**b**) atypical and (**c**) classical MBCs in subjects with acute *P. vivax* infection (AC, n = 9) compared to post-infection (18 months, n = 9) and those of healthy controls (HC, n = 12) were determined by flow cytometric analysis. Activated MBCs (CD10^−^CD19^+^CD20^+^CD21^+^CD27^+^), atypical MBCs (CD10^−^CD19^+^CD20^+^CD21^−^CD27^−^), classical MBCs (CD10^−^CD19^+^CD20^+^CD21^−^CD27^+^)

To characterize the MBC sub-sets in relation to the responses of rhoptry-specific MBCs after *P. vivax* infection, activated, atypical and classical MBCs were determined by phenotyping PBMC samples from both acute phase and at 18 months post-infection. Of the 10 patients in the sub-cohort 2 study, 9 had available PBMCs for MBC sub-set phenotyping by flow cytometric analysis. In acute infection, the frequencies of the activated and atypical MBCs were significantly elevated, whereas classical MBCs were significantly reduced compared to those of healthy controls (Fig. 6a–c). In contrast, activated and atypical MBCs were decreased and classical MBCs increased at 18 months post-infection (activated MBC: average 2.43%, SD 1.32%; atypical MBCs: average 4.37%, SD 1.67%; classical MBCs: average 56.88%, SD 15.34%) (Fig. 6a–c). These data indicated that classical MBCs were the only MBC sub-set which persisted at 18 months post-infection and maintained positive MBCs specific for rhoptry proteins.

## Discussion

It has long been known that individuals living in areas of high malaria endemicity are susceptible to re-infection because their anti-malarial antibodies are short-lived [20, 21]. However, antibody responses to malaria infection develop more effectively in low transmission areas, even after only one or two documented *P. falciparum* infections [18, 19]. Here, two rhoptry proteins of the *P. vivax* parasite, PvRALP1-Ecto and PvRhopH2, were expressed and cloned to explore the persistence of naturally acquired antibody and MBC responses in individuals who lived in an area of low malaria endemicity in southern Thailand, using both cross-sectional and cohort study designs. The IgG antibody responses to PvRALP1-Ecto and PvRhopH2 rhoptry proteins were detected at significant levels during acute vivax malaria, predominantly of IgG1, IgG2 and IgG3 responses but these humoral responses were not maintained after infection. The prevalence of anti-PvRALP1-Ecto and -PvRhopH2 responses fell to 38.89% and 77.78% at 9 months post-infection. In contrast, the analysis of MBC responses to PvRALP1-Ecto and PvRhopH2 showed that positive rhoptry-specific MBCs persisted in a majority of individuals for at least 18 months post-infection. However, it showed no relation of these MBCs with circulating antibody responses, as around 80% of those subjects seronegative for anti-rhoptry antibodies were positive for specific MBCs. These findings suggest that whether the development and persistence of MBCs specific to rhoptry antigens will be important to antibody-based strategies of vaccine development against the blood-stage of *P. vivax* will require further study.

Rhoptry proteins are among the blood-stage antigens that have been characterized and shown to induce antibody responses [14, 25]. However, the majority of previous studies that have assessed antibody responses to rhoptry proteins used cross-sectional study designs to provide information on antibody acquisition and so propose this antibody as a serologic marker of recent exposure to *P. vivax* [44–46]. These studies lacked detailed information regarding antibody longevity and protective immunity against clinical disease, which are important in consideration of potential vaccine candidates. Here, two proteins of schizont rhoptries, PvRALP1-Ecto and PvRhopH2, were expressed to explore their ability to induce long-term antibody responses after natural *P. vivax* exposure. Approximately 66.67 and 74.36% of total patients were found to be seropositive, supporting the high seroprevalence of these antibodies in human sera from areas of Korea, Thailand and Mali [33, 35]. Furthermore, the monitored antibody responses after patient recovery from infection showed significant reduction of antibody levels 9 months post-infection. Some patients maintained these responses until 12 months in the absence of re-infection. Comparing antibody responses to these two proteins, anti-PvRhopH2 responses were more frequent than anti-PvRALP1-Ecto antibody responses with approximately 77.78 and 72.22% of samples seropositive 9 and 12 months post-infection, respectively. Of the 18 patients in the one-year cohort study, only one patient (Pv03) was re-infected with *P. vivax,* whereas the other patients were having their first *P. vivax* infection. Long-term antibody responses to PvRALP1-Ecto and PvRhopH2 proteins could be the main immune component for protection against blood-stage vivax malaria, as previously reported with anti-RALP1, anti-Rhoptry associated protein 1 (RAP1), or anti-RAP2 antibodies that inhibit *P. falciparum* parasite invasion [30, 47, 48]. Moreover, the protective efficacy of anti-PvRAP2 antibody was detected in the non-human *Aotus* primate model by showing the capacity to reduce parasitaemia after rPvRAP2 immunization [46]. However, further study of the antibodies in blocking *P. vivax* parasite invasion, as well as identification of target anti-rhoptry antibodies, will be useful for consideration of these two proteins as potential vaccine candidates.

Analysis of IgG sub-class responses to PvRALP1-Ecto and PvRhopH2 antigens is important for evaluating protective activity since different IgG sub-class responses mediate distinct immune effector functions [42, 43]. Also, greater understanding of the acquisition of natural immunity would strengthen the foundation of vaccine development. IgG1 and IgG3 are considered to be protective antibodies against *Plasmodium* spp infection through complement-mediated lysis [49] and cell-mediated mechanisms, such as opsonic phagocytosis [50–52] and antibody-dependent cellular inhibition (ADCI) [53]. In contrast, the role of IgG2 and IgG4 in protective responses has long been a matter of debate. Some studies show that IgG2 responses are mostly associated with malaria risk [43, 54, 55] whereas a protective role of IgG2 was reported in *P. falciparum* exposure [56–58]. The anti-PfRESA and anti-PfMSP2 responses show potential associations with immunity despite presenting the lowest levels [58]. Here, the pattern of antibody responses to PvRALP1-Ecto and PvRhopH2 proteins were IgG1, IgG2 and IgG3 during acute malaria. Interestingly, IgG1 and IgG2 responses against these two proteins were stably maintained at post-infection, as shown by the 18 months post-infection seropositivity, whereas anti-PvRALP1-Ecto and -RhopH2-IgG3 responses did not persist. This could be because the half-life of IgG3 appears shorter than that of other IgG sub-classes [59]. Consistence with the results of other studies, blood-stage antigens of both *P. vivax* and *P. falciparum* have the ability to trigger B cells to produce cytophilic IgG1 and IgG3 [60–62] and non-cytophilic IgG2 responses [38, 60, 63]. In the present study, the development and persistence of a mixed IgG1/IgG2 response to PvRALP1-Ecto and PvRhopH2 suggested that rhoptry proteins have some characteristic that could stimulate effector T cells and influence the pattern of IgG isotype switching to IgG1 and IgG2 during acute malaria and after infection. Consequently, it should benefit *P. vivax*-infected patients in killing the parasite. This conclusion comes from previous reports that cytophilic IgG1 has the high affinity to Fcγ receptors [64], thus, when present, it is more effective in mediating effector mechanisms, complement-mediated lysis, opsonic phagocytosis, and antibody-dependent cellular inhibition (ADCI) [49, 50, 52, 53]. In case of IgG2, it inhibits parasite invasion and blocks cyto-adherence of infected RBCs to endothelial cells, as well as activates effector cells through FcγRIIa for cell-mediated immunity [57, 65]. However, future studies are required to investigate in depth how these two IgG isotype responses to PvRALP1-Ecto and PvRhopH2 block parasite binding and invasion of red blood cells. Studies are also needed to assess the relationship between anti-rhoptry IgG1 and IgG2 antibody titres and levels of parasitaemia, degrees of prior exposure and rates of re-infection with *P. vivax.*

MBCs are major contributors to antibody production, but it is unclear what factors interfere with or promote maintenance of long-lived antibodies. Here, the presence of rhoptry-specific MBCs in individuals with natural *P. vivax* infections was reported. The specific MBC responses to PvRALP1-Ecto and PvRhopH2 antigens were detected at high frequency at 18 months post-infection (approximately 70 and 90% of subjects). These findings suggest the ability of PvRALP1-Ecto and PvRhopH2 antigens to induce MBC development during malaria infection, and their persistence in the absence of additional malaria antigen stimulation. The literature on MBCs to malaria is based on populations with various intensities of transmission. The *P. vivax* subjects who live in low malaria transmission areas of northern Thailand and northwestern Brazil appear to maintain MBC responses against malaria-specific antigens for many years after their last infection [18, 19]. In contrast, specific MBCs were observed to be short-lived in individuals who reside in areas with high malaria transmission [20, 21]. However, the longevity of PvRALP1-Ecto and PvRhopH2-specific MBCs appears unrelated to the duration of antibody responses, as 85.71% (6 of 7) and 66.67% (6 of 9) of our vivax subjects who had long-lived MBC responses were seronegative to the respective antigen at follow up. These results suggest that circulating antibodies are likely associated with underestimates of the presence of specific MBCs in natural *P. vivax* infections. One explanation is that the alterations of circulating MBC sub-sets, including the expansion of atypical MBC sub-sets, could interfere with the development of long-lived antibody secreting cells and thus antibody production as this MBC sub-set in *P. falciparum* exposure expressed lower levels of surface IgG, possessed reduced B cell receptor signalling, and impaired antibody production in vitro [66, 67]. However, to date, the functional significance of atypical MBC expansion in the acquisition and persistence of humoral immunity to malaria, as well as the factors that generate and maintain antibody-secreting cells to infections, remain unclear. Further studies to unravel a relationship between atypical MBCs and immune protection or disease progression in malaria infection are needed.

An alteration of circulating MBC populations was detected during malaria infection [40, 68, 69]. Here, activated and atypical MBCs were identified as the MBC sub-sets that played major roles in the responses to rhoptry proteins during acute vivax episodes. However, by 18 months post-infection these sub-sets had decreased and classical sub-sets increased. To explain these finding, it is possible that CD27^+^ classical MBCs were triggered via parasite infection, and class-switched into CD27^+^ activated MBCs or CD27^−^ atypical MBCs, helping in parasite clearance. After infection these two MBC sub-classes were reduced and class-switched back to CD27^+^ classical MBCs. However, the relationship these MBC sub-sets to plasmablast/plasma cell responses, as well as that between long-lived plasma cells and antibody longevity in recovered subjects (18 months post-infection) was not demonstrated in this study. A full understanding of these relationships would be useful for the context of immunity to malaria infection and vaccine development, since generation of pre-existing antibody from long-lived plasma cells could help to kill parasites. In accordance with the previous studies, the temporary changes in MBC sub-sets was previously reported from an area with seasonal malaria transmission [40, 68, 69]. There, malaria-specific antibodies and MBCs were sustained whereas the cumulative exposure to malaria gradually altered the delicate balance between circulating classical MBCs and functionally impaired atypical MBCs [69]. Despite a negative impact of atypical MBCs on the efficacy of malaria vaccines, a rare study did detect malaria-specific atypical MBCs and observed their function in natural exposure [70]. Most of MBC sub-set studies reported total populations which phenotyped as CD19^+^CD21^−^CD27^−^ (atypical MBCs) or CD19^+^CD27^+^ (classical MBCs), without any malaria specificity [40, 68, 69]. Further studies are needed to generate a high sensitivity technique for detecting the rare populations of each malaria-specific MBC sub-set, and to study the function of atypical MBCs and their relationship to classical MBCs in circulating blood.

The limitations of this study include the lack of an independent measure of prior *P. vivax* and *P. falciparum* exposure that would be involved in the persistence of antibody and MBC responses. The study was also limited by the inability to recruit a larger group of *P. vivax* subjects into the cohort study. (Only 18 and 10 patients were able to be followed at 12 and 18 months in the sub-cohort 1 and 2 studies, respectively.) The observation in this study that showed persistence of long-lived PvRALP1-Ecto- and PvRhopH2-specific MBCs in the absence of re-infection should be confirmed by future studies. In addition, a larger sample size would allow correlation analyses among anti-PvRALP1-Ecto and -PvRhopH2 levels, specific MBCs and frequency of MBC sub-sets.

## Conclusions

The results in this study provide additional information to the available data on the characteristics of PvRALP1-Ecto and PvRhopH2 proteins, and their ability to induce humoral immunity during acute malaria and after infection. The anti-PvRALP1-Ecto and -PvRhopH2 responses in the majority of individuals were short-lasting after infection. However, the specific memory B cells in a sub-group of individuals was stably detected (through 18 months). This evidence supports previous reports that effective long-lived MBCs can develop and persist in regions with low rates of malaria transmission.

## Supplementary information

**Additional file 1.** PvRALP1-Ecto and PvRhopH2 specific MBC response by ELISPOT assay.

## Data Availability

The datasets used and/or analysed during the current study are available from corresponding author upon reasonable request.

## Abbreviations

MBCs: memory B cells
ASC: antibody-secreting cells
RALP1: rhoptry-associated leucine zipper-like protein 1
RhopH: high-molecular weight complex rhoptry proteins
RBC: red blood cells
