# Supplementary material for: The persistence of naturally acquired antibodies and memory B cells specific to rhoptry proteins of Plasmodium vivax in patients from areas of low malaria transmission
Source: Malar J. 2019 Nov 29;18:382. doi: 10.1186/s12936-019-3009-2 (PMC6884809; doi:10.1186/s12936-019-3009-2)
Supplement: Supplementary file 1 — Additional file 1. PvRALP1-Ecto and PvRhopH2 specific MBC response by ELISPOT assay. [file 12936_2019_3009_MOESM1_ESM.docx]

**
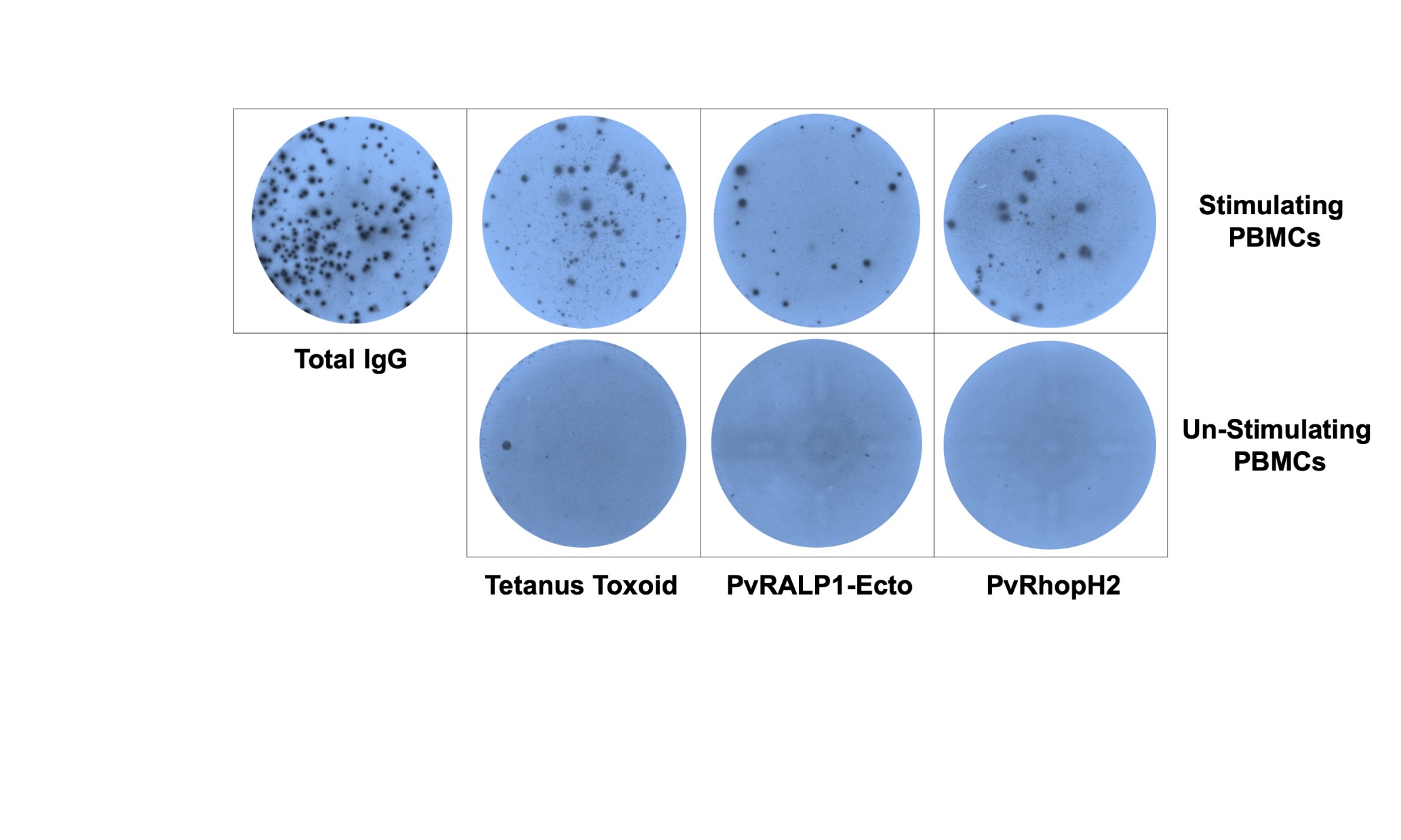
**

Additional file 1: PvRALP1-Ecto and PvRhopH2 specific MBC response by ELISPOT assay. The numbers of specific MBCs produced in response to the PvRALP1-Ecto, PvRhopH2, tetanus toxoid and total IgG in individual PBMCs that persisted 18 months after the *P. vivax* infection were determined using an ELISPOT assay (n=10). For the analysis of antigen specific MBCs responses, PBMCs were cultured with or without stimulation of R848 and recombinant human IL-2. After that, cells were harvested and applied to ELISPOT assay.
